# SAMHD1 … and Viral Ways around It

**DOI:** 10.3390/v13030395

**Published:** 2021-03-02

**Authors:** Janina Deutschmann, Thomas Gramberg

**Affiliations:** Institute of Clinical and Molecular Virology, Friedrich-Alexander University Erlangen-Nürnberg, 91054 Erlangen, Germany; janina.deutschmann@uk-erlangen.de

**Keywords:** SAMHD1, restriction factor, viral antagonism, HIV, herpesviruses, viral kinases, Vpx, dNTP hydrolase, viral interference

## Abstract

The SAM and HD domain-containing protein 1 (SAMHD1) is a dNTP triphosphohydrolase that plays a crucial role for a variety of different cellular functions. Besides balancing intracellular dNTP concentrations, facilitating DNA damage repair, and dampening excessive immune responses, SAMHD1 has been shown to act as a major restriction factor against various virus species. In addition to its well-described activity against retroviruses such as HIV-1, SAMHD1 has been identified to reduce the infectivity of different DNA viruses such as the herpesviruses CMV and EBV, the poxvirus VACV, or the hepadnavirus HBV. While some viruses are efficiently restricted by SAMHD1, others have developed evasion mechanisms that antagonize the antiviral activity of SAMHD1. Within this review, we summarize the different cellular functions of SAMHD1 and highlight the countermeasures viruses have evolved to neutralize the restriction factor SAMHD1.

## 1. The dNTPase SAMHD1

**Figure 1:**
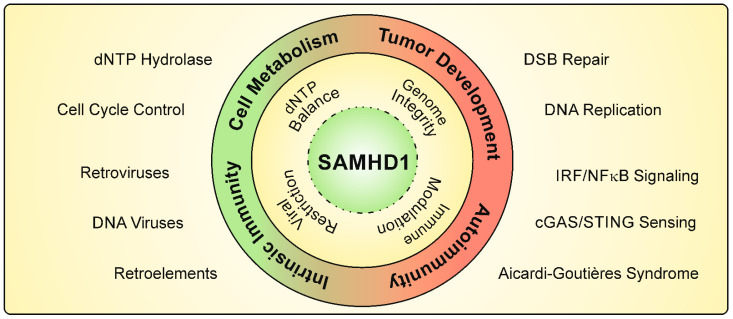
Cellular functions of SAMHD1. The dNTP triphosphohydrolase SAMHD1 is an important regulator of dNTP levels. A correct balance of the cellular dNTP pool has been shown to be important for cell-cycle control and genome integrity. SAMHD1 is a mediator of double-strand break (DSB) repair and ensures the progression of DNA replication. Mutations in the *SAMHD1* gene have been identified in various types of cancer. Furthermore, SAMHD1 potently restricts viral infectivity of retroviruses and DNA viruses, and blocks the activity of endogenous retroelements. Mutations in *SAMHD1* are correlated with autoimmune diseases such as Aicardi–Goutières syndrome, resulting in elevated type I IFN levels. SAMHD1 also impairs the innate immune sensing of viral infections by interfering with cGAS/STING nucleic acid recognition and NFκB/IRF signaling pathways.

The SAM and HD domain-containing protein 1 (SAMHD1) is a ubiquitously expressed deoxynucleotide triphosphohydrolase (dNTPase) of 626 amino acids (Figure 1). In general, sterile alpha motif (SAM) domains have been shown to mediate protein–protein interaction or nucleic acid binding; however, its function in SAMHD1 is still unclear. The enzymatically active HD domain, defined by two pairs of histidine and aspartate residues in its active center, on the other hand is essential for retroviral restriction and tetramerization of the protein [1,2,3]. Although nuclear localization of SAMHD1 is mediated through an N-terminal nuclear localization signal (NLS), neither the antiviral activity nor the dNTPase function of SAMHD1 seem to be influenced by its localization [4,5,6]. Oligomerization of SAMHD1 is required for its catalytic dNTPase activity and is induced upon cofactor binding at two allosteric sites within the protein [7,8,9,10,11,12]. At allosteric site 1, dGTP or GTP binding leads to SAMHD1 dimerization, while the subsequent binding of any dNTP to allosteric site 2 induces tetramer formation [1,13,14].

SAMHD1 converts dNTPs into deoxynucleosides (dN) and inorganic triphosphates (PPPi) and thereby counteracts the de novo dNTP synthesis, primarily conducted by ribonucleotide reductase (RNR) and cellular deoxynucleoside kinases, which are mainly active during the S phase of the cell cycle [15,16,17,18]. RNR catalyzes the formation of dNTPs from ribonucleotides, while deoxynucleoside kinases, such as thymidine kinases (TK), add phosphates to nucleosides to form deoxynucleoside triphosphates [19,20]. This ensures a highly balanced dNTP pool during the progression of the cell cycle, with a sufficient supply of dNTPs for efficient genome replication in dividing cells and limited levels of dNTPs in nondividing and resting cells [1,14,21]. The cyclin-dependent kinases (CDK) 1 and 2, together with cyclin A, phosphorylate SAMHD1 at the threonine residue 592 (T592) during the S and G_2_ phase of the cell cycle [22,23]. SAMHD1 is dephosphorylated during the M/G1 phase transition by the PP2A-B55α phosphatase and is dephosphorylated in the G_0_ and G_1_ phases [24]. Thus, SAMHD1 is phosphorylated during the progression of the cell cycle, which correlates with the demand of cellular dNTPs. However, at this point it is unclear whether the phosphorylation at T592 indeed regulates the dNTPase activity of SAMHD1 [25,26,27,28].

## 2. DNA Replication and DNA Damage Repair

By degrading cellular dNTPs, SAMHD1 is a major regulator of nucleotide homeostasis. A highly balanced dNTP pool is essential for genomic integrity, including proper DNA replication and efficient repair of DNA breaks. An imbalance can lead to a deregulated cell-cycle progression and therefore induce replication stress, which might eventually result in the accumulation of genomic mutations [29,30]. During host genome replication, SAMHD1 was found to be recruited to replication foci to regulate the progression of the replication fork. Here, SAMHD1 activates the 3′-5′-exonuclease MRE11, thereby promoting the MRE11-mediated degradation of nascent DNA at stalled replication forks, which has been suggested to prevent the induction of type I interferons (IFN) through the aberrant accumulation of single-stranded DNA (ssDNA). In addition, stimulation of MRE11 by SAMHD1 leads to activation of the ATR-CHK1 checkpoint, resulting in restart and progression of stalled replication forks [31].

SAMHD1 has also been described to be important for DNA damage repair by promoting homologous recombination (HR) [32]. SAMHD1 was identified to localize to DNA double-strand breaks (DSBs) and to recruit members of the DSB repair (DSBR) machinery [32,33]. It directly binds to the C-terminal binding protein interacting protein (CtIP), leading to its recruitment to DSBs and consequently to the activation of MRE11 as part of the MRE11-Rad50-NBS1 (MRN) DSBR complex, which is responsible for the resection of DNA ends via the 3′-5′-exonuclease function of MRE11 [32,33]. Due to its role in DSBR, it is conceivable that mutations in the *SAMHD1* gene, as well as silencing hypermethylation of the *SAMHD1* promoter, might play a role in tumorigenesis of various forms of cancer such as colon cancer, chronic lymphocytic leukemia, and lung adenocarcinoma [30,34,35,36].

## 3. Role of SAMHD1 in Intrinsic Immunity

Mutations in *SAMHD1* are associated with Aicardi–Goutières syndrome (AGS)—an autoimmune disorder characterized by a progressive inflammatory encephalopathy, spontaneous type I IFN production in the cerebrospinal fluid, and upregulated IFN-stimulated gene (ISG) expression [6]. AGS highly resembles the phenotype of congenital viral infections, with signs of neurological dysfunction, physical and mental retardation, and basal ganglia calcification [37,38]. The development of AGS is linked to mutations in several genes involved in nucleic acid metabolism or sensing, including ADAR1, TREX1, RNaseH2, MDA5, and *SAMHD1* [6,37,39]. Defects or alterations in these genes can lead to enhanced accumulation or sensing of endogenous nucleic acids, which in turn drive inflammatory immune responses in AGS patients [6,38,40]. In line with its role in AGS, depletion of SAMHD1 results in a highly elevated type I IFN production in human macrophages, as well as in an upregulation of ISGs in the mouse model [41,42,43].

Besides its protective role in autoimmunity, SAMHD1 was also shown to suppress the antiviral immune response upon infection [44]. HIV-1 reverse-transcription products have been shown to be recognized by the pattern recognition receptor cyclic GMP-AMP synthase (cGAS) in myeloid cell lines such as dendritic cells (DC) [45,46,47]. Stimulation of cGAS, and subsequently of the adaptor protein STING, leads to the activation and nuclear translocation of the transcription factors nuclear factor κB (NFκB) and IFN regulatory factor 3 (IRF3), culminating in the production of proinflammatory cytokines [44,48]. Interestingly and somewhat counterintuitive, SAMHD1 restricts reverse transcription of the HIV-1 genome in nondividing cells, and therefore dampens the sensing of HIV DNA products by cGAS in myeloid cells such as DCs [45,46,47]. In addition, SAMHD1 inhibits the activation of NFκB and type I IFN upon viral infection through direct binding to NFκB as well as IRF7 [49]. Furthermore, SAMHD1 reduces the phosphorylation of NFκB inhibitor IκBα, and interacts with the inhibitor κB kinase ε (IKKε), which prevents IKKε-dependent phosphorylation and activation of IRF7 [49]. Thus, SAMHD1 interferes with several mediators of immune signaling pathways and prevents an excessive antiviral and proinflammatory response with a secretion of cytokines such as tumor necrosis factor α (TNFα), interleukin 1β and -6 (IL-1β/-6), IFN α and β induction, resulting in a downregulated recruitment of immune cells [44,49,50,51,52].

## 4. The Antiviral Activity of SAMHD1

### 4.1. Retroviruses

A hallmark of the retroviral life cycle is the reverse transcription of the viral RNA genome, followed by integration of the cDNA into the host genome in form of proviral DNA. Thus, retroviruses require relatively high intracellular dNTP levels for reverse transcription. In nondividing cells, SAMHD1 limits viral infectivity by downregulating cellular dNTP concentrations required for an efficient reverse transcriptase (RT) activity [53]. Initially, SAMHD1 has been identified as a potent restriction factor against HIV-1 in nondividing myeloid cells, such as macrophages and DCs, and resting CD4^+^ T cells [54,55,56,57]. Experiments in mice revealed that SAMHD1 also inhibits pseudotyped single-cycle HIV-1 reporter virus infection in vivo [41,42]. While SAMHD1 is dephosphorylated and antiviral-active in nondividing cells, CDK1/2-dependent phosphorylation of the regulatory residue T592 in cycling cells was found to abolish its activity against HIV-1 [22,25]. However, whether the dNTP hydrolase function of SAMHD1 is also regulated by T592 phosphorylation is not clear [25,26,27,28]. Of note, a ribonuclease (RNase) activity was described for SAMHD1 by two groups, suggesting that SAMHD1 might directly degrade retroviral genomic RNA upon entry [58,59]. However, other groups refuted the findings and found that SAMHD1, despite its RNA-binding capabilities, does not contain an RNase activity, which, according to Seamon and colleagues, originates from endogenous nucleases easily coprecipitated with recombinant SAMHD1 [60,61].

In addition to HIV-1, SAMHD1 has been shown to inhibit other lentiviruses, including HIV-2, several simian immunodeficiency viruses (SIV), feline immunodeficiency virus (FIV), bovine immunodeficiency virus (BIV), and equine infectious anemia virus (EIAV) [2,62]. SAMHD1 also downregulates the infectivity of other retroviruses like the β-retrovirus Mason–Pfizer monkey virus (MPMV) and the α-retrovirus Rous sarcoma virus (RSV) in myeloid cells [2,62]. Sze and colleagues reported that infection of human T cell leukemia virus 1 (HTLV-1) was abrogated in primary macrophages by SAMHD1 through activation of a STING-mediated apoptosis pathway [63]. Moreover, reverse transcription of the γ-retroviruses N- and B-tropic murine leukemia viruses (MLV) was potently abolished in differentiated myeloid cell lines expressing exogenous SAMHD1, as well as in primary monocyte-derived macrophages (MDM) [2,62].

Together, these findings show that the SAMHD1-mediated restriction is not limited to HIV and SIVs, but acts on many different retroviruses upon entry of nondividing cells, with the notable exception of foamy viruses, which enter new target cells with an almost completely reverse-transcribed viral genome [62]. Another exception became obvious in the SAMHD1 knockout (KO) mouse model, in which viral infectivity of the Friend leukemia virus (FV) was found to be unaffected by the presence of SAMHD1 [42]. However, since the main target cells of FV are dividing erythroid progenitor cells, in which SAMHD1 is not antiviral-active, it is conceivable that FV replication is not targeted by SAMHD1 in vivo [42].

### 4.2. Retroelements

In addition to exogenous retroviruses, SAMHD1 has also been shown to downregulate the activity of endogenous retroelements. In 2013, Zhao and colleagues found that SAMHD1 inhibits the retrotransposition of long interspersed nuclear element 1 (LINE-1) GFP-reporter elements and identified AGS-associated SAMHD1 mutants to be defective for LINE-1 restriction [64]. Although the anti-LINE-1 activity was confirmed by two independent groups, the exact mechanism of restriction remains unclear [65,66].

Interestingly, however, all groups found that SAMHD1 is active against LINE-1 retroelements in cycling cells. In addition, Herrmann et al. found that the capacity of SAMHD1 to block the activity of LINE-1 was regulated by phosphorylation of T592 but did not correlate with its dNTPase activity [66]. Together, these findings suggest that the mechanism of LINE-1 inhibition by SAMHD1 is similar but not identical to HIV-1 restriction and that endogenous LINE-1 elements might play a role in SAMHD1-related AGS and tumorigenesis in the presence of defective SAMHD1.

### 4.3. DNA Viruses

DNA viruses also rely on cellular dNTPs for replication of their genome. Consequently, several DNA viruses were found to be negatively affected by the dNTPase activity of SAMHD1. Hepatitis B virus (HBV) belongs to the hepadnavirus family and replicates its viral DNA genome through an RNA intermediate and a reverse-transcription step. In 2014, Chen et al. found that SAMHD1 potently restricts the viral expression and replication of transfected HBV DNA in liver cell lines [67]. The authors showed that the catalytic-inactive SAMHD1-HD/AA mutant also restricts HBV antigen expression, indicating that SAMHD1 inhibits HBV by a different mechanism than retroviruses. However, later studies contradicted this work and found a potent dNTP hydrolase activity of SAMHD1 to be essential for restriction of HBV DNA synthesis [68,69]. Another virus affected by SAMHD1 is human papillomavirus 16 (HPV16). Its replication has been shown to be enhanced in absence of SAMHD1, leading to cell hyperproliferation of keratinocytes infected with HPV16 [70].

SAMHD1 was also found to potently inhibit the infection of vaccinia virus (VACV), a member of the poxvirus family, and the α-herpesvirus herpes simplex virus 1 (HSV-1) by limiting intracellular dNTP levels through its dNTP hydrolase activity in nondividing myeloid cell lines [71,72]. Recently, SAMHD1 was shown to restrict the β-herpesviruses human cytomegalovirus (HCMV), murine cytomegalovirus (MCMV), as well as the γ-herpesvirus Epstein–Barr virus (EBV) [73,74,75,76]. Businger and colleagues found that HCMV replication is efficiently restricted by SAMHD1 in primary MDMs [73]. Furthermore, Kim et al. demonstrated that SAMHD1 inhibits the HCMV immediate early (IE) gene expression by direct binding to NFκB [76]. The major immediate early (MIE) promoter contains several NFκB and AP-1 binding sites for induction of IE gene transcription, which is blocked by SAMHD1 binding to NFκB and IKKε [49,76,77]. In line with these results, we found that the replication of MCMV is restricted by murine SAMHD1 in vivo [74]. Infection of WT and SAMHD1 KO mice revealed an enhanced replication of MCMV in SAMHD1 KO mice, showing for the first time that SAMHD1 acts as an antiviral restriction factor against a replicating virus in vivo. Moreover, SAMHD1 depletion in latently EBV-infected Akata cells resulted in an increased accumulation of viral particles upon EBV reactivation, suggesting that SAMHD1 also inhibits EBV replication [75].

## 5. Viral Antagonisms to SAMHD1

**Figure 2:**
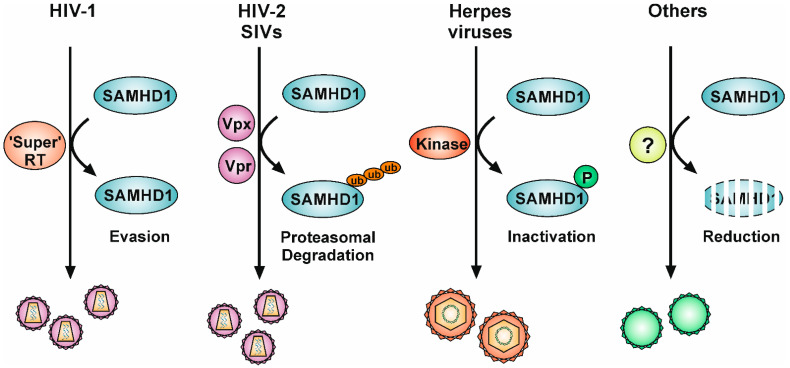
Viral countermeasures to circumvent the intrinsic restriction factor SAMHD1. Retroviruses without a Vpx-like function, such as HIV-1, encode a highly efficient reverse transcriptase (“Super” RT) that enables the virus to reverse-transcribe its genome despite the presence of active SAMHD1 and the resulting low dNTP level. HIV-2 and related SIVs encode the small accessory protein Vpx (or Vpr with a Vpx-like function), which ties SAMHD1 to a Cullin E3 ubiquitin ligase complex, resulting in ubiquitination and subsequent proteasomal degradation of SAMHD1. Herpesviruses, especially β- and γ-herpesviruses, rely on their conserved serine/threonine protein kinases to phosphorylate and thereby inactivate SAMHD1. In addition, HCMV infection also induces the proteasomal degradation of SAMHD1. Infection with other viruses, such as HSV-2, HBV, and HPV16, has been reported to cause a reduction in SAMHD1 RNA or protein levels, suggesting that additional unknown mechanisms exist to circumvent the SAMHD1-mediated restriction. Ub: ubiquitination, P: phosphorylation.

While SAMHD1 experienced positive selection during host–pathogen coevolution, several viruses have also developed evasion strategies to antagonize the restriction by SAMHD1 [78,79,80]. Thus, the following chapter will focus on the different countermeasures that viruses have evolved to circumvent the restrictive activity of SAMHD1 (Figure 2; Table 1).

### 5.1. The Viral Accessory Proteins Vpx and Vpr

A hallmark of lentiviral genomes is the presence of small accessory open reading frames. While both HIV-1 and HIV-2 encode the accessory protein Vpr, only HIV-2 and related SIVs contain an additional open reading frame for Vpx [81,82]. Vpx arose from Vpr by an ancient gene duplication event in a lentiviral precursor virus, suggesting two similar but not identical functions in both proteins [83]. SIV from sooty mangabeys (SIVsmm) is the zoonotic precursor of HIV-2 and gave rise to SIV found in macaques in captivity (SIVmac). Thus, Vpx and Vpr proteins of these viruses share a high sequence homology. SIVsmm infection of pigtailed macaques revealed that Vpx and Vpr are important for dissemination and pathogenesis of the virus in vivo [84]. Both Vpx and Vpr are actively packaged into virions by binding to the p6 domain of the Gag polyprotein and were therefore thought to play an important role during the early steps of infection, prior to integration into the host genome [85]. Vpx, but not Vpr, is critical for the ability of HIV-2/SIVsmm to efficiently infect monocytes, macrophages, and DCs [86,87,88]. Thus, it has been suggested that Vpx inactivates an early post-entry block in these cells. Indeed, in 2011, Vpx of HIV-2/SIVsmm was shown to counteract the HIV-1 restriction factor SAMHD1 in myeloid cells and resting CD4^+^ T cells [54,55,56,89]. Interestingly, Vpx is not only enhancing SIV infectivity, but also boosts the infection of different retroviruses, including HIV-1, in *trans*, when delivered into cells by virus-like particles [2,62,90]. In target cells, Vpx induces the proteasomal degradation of SAMHD1 by tying it to the ubiquitin–proteasome system [54,55]. Cullin E3 ubiquitin ligase complexes consist of a central Cullin scaffold protein, a catalytic RING subunit, and varying adapter proteins mediating interaction with specific target proteins [91]. In case of Vpx and Vpr, coisolation approaches revealed that both proteins interact with a Cullin4 E3 ubiquitin ligase complex via the adapter proteins damage-specific DNA binding protein 1 (DDB1) and DDB1 Cullin-associated factor 1 (DCAF1) [92,93,94,95,96,97,98]. By binding to SAMHD1, Vpx and some SIV Vpr proteins render SAMHD1 a substrate for polyubiquitination and thereby induce its proteasomal degradation.

The genetic conflict between innate immunity and viruses results in the rapid selection of mutations in proteins involved in direct host–pathogen interaction. The groups of Monsef Benkirane and Michael Emerman described this “arms race” for SAMHD1 and Vpx/Vpr [83,99]. While Vpr is expressed by all primate lentiviruses, a *Vpx* gene is only present in two out of eight SIV lineages, the HIV-2/SIVsmm/SIVmac lineage and the SIV rcm/SIVmnd2 (SIV of red-capped mangabeys, SIV of mandrill) lineage. Lim and colleagues analyzed SAMHD1 degradation upon cotransfection with Vpr and Vpx proteins from different SIVs [83]. While Vpx from SIVmac did degrade human and all simian SAMHD1 proteins tested, other Vpx proteins only counteracted SAMHD1 from certain species. In a similar approach, Laguette and colleagues demonstrated that the interaction of Vpx and SAMHD1 is species-specific [99]. Both studies also identified specific SIV Vpr proteins that were able to degrade SAMHD1. These Vpr proteins are found in viruses lacking Vpx, and belong to the two SIV lineages that include SIV infecting African green monkeys (SIVagm) or Sykes monkeys (SIVsyk) and greater spot-nosed monkeys (SIVgsn). Comparative analysis showed that the neofunctionalization of Vpr to degrade SAMHD1 has occurred once in an ancient predecessor and, importantly, prior to the gene duplication or recombination event that gave rise to Vpx. This suggests that viruses with an additional Vpx reading frame had an evolutionary advantage, perhaps driven by positive selection events in SAMHD1. Indeed, both studies showed that the antagonism by Vpx and Vpr proteins drove positive selection of SAMHD1 in Old World monkeys. Sites of strong positive selection can be found in the N-terminal and C-terminal regions of SAMHD1. Fregoso et al. determined that different Vpx and Vpr proteins have evolved to recognize distinct N- or C-terminal interfaces of SAMHD1, which is in line with the idea of a rapidly evolving SAMHD1 [100]. Schwefel and colleagues were able to corroborate these findings and solved the crystal structures of SIV Vpx of the mandrill lineage together with the N-terminus of SAMHD1 and DCAF1, as well as SIVsmm together with the C-terminus of SAMHD1 and DCAF1 [101,102].

Interestingly, another lentivirus, EIAV, has evolved a different strategy to circumvent SAMHD1 restriction in equine macrophages [103]. While equine SAMHD1 restricts replication at the reverse-transcription step, EIAV seems to employ its transcriptional regulator Rev to counteract SAMHD1 by downregulating the equine SAMHD1 level via a lysosomal pathway to ensure efficient EIAV replication [103].

### 5.2. “Super-RT”: Highly Efficient Lentiviral Reverse Transcriptases

Nondividing myeloid cells, such as macrophages or microglia, are targeted by many lentiviruses during the course of infection. However, not all lentiviruses encode Vpx, or a protein with a Vpx-like function, to inactivate SAMHD1. Instead, to overcome the SAMHD1-driven shortage of dNTPs in nondividing cells, viruses lacking Vpx harbor very efficient reverse transcriptases that polymerize viral cDNA at low dNTP concentrations [104,105,106,107]. For instance, Lenzi and colleagues biochemically characterized RTs from HIV-1, HIV-2, and SIV isolates and determined the K_m_ values of HIV-1 RTs to be very low in general and in the range of the low dNTP concentrations found in nondividing macrophages. In contrast, enzymes encoded by viruses that trigger SAMHD1 degradation, such as HIV-2, displayed significantly higher K_m_ values and required higher dNTP concentrations to efficiently generate cDNA [104]. Interestingly, the Kim laboratory also cloned and analyzed the sequences of RT genes derived from Rhesus macaques infected with either SIVmac WT virus encoding Vpx or a genetically engineered variant lacking the accessory protein [106]. The authors compared RT sequences isolated from these monkeys at 6 to 7 months postinfection (mpi) and at 36 mpi, and found an enhanced accumulation of mutations in RT genes from samples infected with virus lacking Vpx. Biochemical analysis revealed that at 36 mpi, RT variants from (−)Vpx viruses displayed higher catalytic and nucleotide incorporation efficacies compared to RTs from (+)Vpx virus-infected animals or compared to samples from early time points [106]. These findings corroborate the idea that lentiviruses without a Vpx-like function may have evolved more efficient RT molecules to enable cDNA synthesis in nondividing cells.

Interestingly, however, pretreatment of nondividing macrophages with Vpx-containing virus-like particles further boosts HIV-1 infectivity in macrophages in vitro, despite optimized RT enzymes [87,108]. At this stage, it is still unclear why HIV-1 and other lentiviruses do not encode a Vpx-like function to degrade SAMHD1 in order to increase infectivity in macrophages. One intriguing idea is that the advantage to infect macrophages more efficiently by degrading SAMHD1 may be opposed in vivo by a simultaneously enhanced infection of DCs, resulting in a stronger antiviral immune response [46,47].

### 5.3. Conserved Herpesviral Protein Kinases

Human herpesviruses (HHV) encode conserved herpesvirus protein kinases (CHPK). This family of serine/threonine kinases includes UL13 of HSV-1 and -2, ORF47 of varicella-zoster virus (VZV), UL97 of HCMV, U69 of HHV-6 and -7, BGLF4 of EBV, and ORF36 of Kaposi’s sarcoma-associated herpesvirus (KSHV) [109,110,111]. These kinases are also referred to as viral CDKs (v-CDKs), since they structurally resemble cellular CDKs and target many of their substrates to modulate cell-cycle-dependent processes to favor viral replication [110,112,113].

In 2019, Kim et al. and Businger et al. analyzed the role of SAMHD1 in HCMV infection of myeloid cell lines [73,76]. They found that SAMHD1 inhibits HCMV gene expression as well as viral genome replication, respectively. In addition, Businger and colleagues observed that the SAMHD1-mediated restriction of HCMV is potently antagonized by the viral kinase pUL97, as well as by activation of cellular CDKs following HCMV infection. SAMHD1 was shown to be strongly phosphorylated at the regulatory T592 site upon HCMV infection, as well as upon transient pUL97 coexpression [73]. Of note, Kim et al. and Chen and colleagues found that the inhibitory effect of SAMHD1 on NFκB-mediated MIE promoter activation and innate immune signaling is independent of T592 phosphorylation, even though phosphorylation is induced upon HCMV infection [49,76]. In an affinity purification and mass spectrometry approach, Bogdanow et al. confirmed SAMHD1 as a target of HCMV pUL97 and of the homologous kinase U69 of HHV7 [114]. In addition, De Meo et al. substantiated previous findings that SAMHD1 is also phosphorylated through induction of CDK2 upon HCMV infection [115].

The murine homolog of SAMHD1 occurs in two isoforms. Isoform 2, which is expressed at low levels, is differentially spliced, and therefore lacks the regulatory C-terminal phosphorylation site. Isoform 1, however, highly resembles the human protein with an equally abundant expression pattern [42,116,117,118,119]. The antiretroviral activity of isoform 1 is negatively regulated by phosphorylation at threonine residue 603 (T603), which corresponds to T592 in human SAMHD1 [42,118,119]. Recently, we found that also the MCMV orthologue of pUL97—M97—strongly phosphorylates murine SAMHD1 at T603 upon infection of murine macrophages, causing the loss of its antiviral activity [74]. In addition, infection with MCMV WT but not with an M97 kinase-deficient (KD) virus significantly increased intracellular dNTP concentrations. Importantly, our study revealed that SAMHD1 restricts MCMV genome replication in vivo and that WT virus replication, and more pronounced M97 KD virus replication, is enhanced in SAMHD1 KO mice [74].

In line with these findings, a recent study by Zhang and colleagues found that the EBV kinase BGLF4 phosphorylates human SAMHD1 at the T592 site. The phosphorylation abrogated EBV inhibition by SAMHD1 as well as its dNTPase activity [75]. In their manuscript, Zhang et al. also analyzed SAMHD1 phosphorylation upon coexpression of other herpesviral CHPKs. The authors found that all kinases of β- and γ-herpesviruses phosphorylate SAMHD1 at T592, while the α-herpesviral kinases UL13 of HSV-1 and -2 and ORF47 of VZV do not affect SAMHD1 phosphorylation [75]. Of note, α-herpesviruses encode an additional serine/threonine kinase, which might inactivate SAMHD1 in these viruses instead of the CHPKs [112,120]. However, it remains unclear whether SAMHD1 phosphorylation plays a role in α-herpesviral infection at all, since Kim and colleagues found that the phosphorylation of SAMHD1 upon infection does not affect the SAMHD1-mediated restriction of HSV-1 [72]. Another interesting feature of several herpesviruses is that they encode viral proteins supporting de novo dNTP synthesis. All members of the α- and γ-herpesviruses express their own RNR and TK proteins, which drive dNTP anabolism and therefore antagonize SAMHD1 activity [121,122,123]. This suggests that these viruses are not as dependent on a kinase-mediated inactivation of SAMHD1 as β-herpesviruses. Of note, also other viruses have developed mechanisms to antagonize the dNTP-catabolizing activity of SAMHD1. VACV encodes its own TK, and Hollenbaugh et al. found that the deletion of TK enhances the inhibitory activity of SAMHD1 in VACV infection [71]. Another example is HBV, which was shown to induce the expression of the cellular RNR subunit R2 to ensure a sufficient dNTP supply upon infection [124,125].

### 5.4. Downregulation of Expression Levels and Protein Relocalization

Aside from the phosphorylation-dependent inactivation of SAMHD1, the downregulation of SAMHD1 expression levels has been observed as a viral countermeasure mediated by several DNA viruses. In case of HCMV, Businger et al. found that the infection of primary MDMs results in a decreased steady state expression of SAMHD1 [73]. This is in line with results from Cheung et al., who reported the expression of several antiviral restriction factors, among them SAMHD1, to be reduced upon infection of CD34^+^ hematopoietic progenitor cells [126]. Hyeon and colleagues also analyzed SAMHD1 protein level and found that HCMV infection induces the proteasomal degradation of SAMHD1 via a Cullin2 E3 ubiquitin ligase complex [127]. In addition, De Meo et al. found that the HCMV-induced phosphorylation of SAMHD1 leads to its cytosolic relocalization and suggested that this relocalization might represent a novel evasion mechanism by HCMV [115]. Of note, the innate DNA sensor and HCMV inhibitory factor IFI16 was also shown to be phosphorylated by pUL97, leading to its inactivation and relocalization in a similar manner [128,129]. Also, infection with HSV-2 has been shown to reduce the expression levels of antiviral restriction factors, including SAMHD1, in DCs [130]. In case of HBV, Chen et al. described a reduced expression of SAMHD1 upon infection of hepatocytes, which could not be confirmed by other groups [67,69]. Also in the context of HPV-16 infection, SAMHD1 RNA as well as protein levels were found to be decreased in infected keratinocytes, suggesting that, similar to other DNA viruses, HPV found a way to circumvent the SAMHD1-mediated restriction in order to efficiently replicate its DNA genome [70].

**Table 1:** Viral SAMHD1 evasion strategies.

	Virus	Mechanism	Antagonism	Reference
**Retroviruses**	HIV-1 (Lenti)	dNTPase	Efficient RT in presence of SAMHD1	[1,14,53,104,105,106,107]
	HIV-2 (Lenti)	dNTPase	Vpx-induced proteasomal degradation	[54,56,90]
	SIV (Lenti)	dNTPase	Vpx/Vpr-induced proteasomal degradation	[54,55,56,57,87,89,90]
	FIV (Lenti)	dNTPase		[2,62]
	BIV (Lenti)	dNTPase		[2]
	EIAV (Lenti)	dNTPase	Rev-induced lysosomal degradation	[2,62,103]
	RSV (α)			[62]
	MPMV (β)			[62]
	HTLV (δ)	STING-mediated apoptosis		[62,63]
	N-/B-MLV, FV (γ)	dNTPase	Replication in dividing cells	[2,42,62]
**Herpesviruses**	HSV-1/2 (α)	dNTPase	SAMHD1 downregulation + viral RNR/TK expression	[71,72,130]
	HCMV (β)	dNTPase, NFκB/IRF inhibition	Phosphorylation by pUL97 + Induction of cellular CDKs + Proteasomal degradation + Cytosolic relocalization	[73,76,115,126,127]
	MCMV (β)	dNTPase	Phosphorylation by M97	[74]
	EBV (γ)	dNTPase	Phosphorylation by BGLF4 + viral RNR/TK expression	[75]
**Others**	Hepadnavirus HBV	dNTPase	SAMHD1 downregulation + RNR induction	[67,68,69,124,125]
	Poxvirus VACV	dNTPase	TK expression	[71]
	Papillomavirus HPV16	dNTPase	SAMHD1 downregulation	[70]
**Retro** **Elements**	LINE-1	ORF2p binding, dNTPase, Sequestering RNPs		[64,65,66]
	Alu/SVA			[64,66]
	IAP/MusD			[66]

## 6. Conclusions

SAMHD1 is a potent restriction factor of various viruses with different replication strategies. The growing spectrum of viruses found to be inhibited by SAMHD1 accentuates its importance for the intrinsic immune defense. During mammalian evolution, SAMHD1 has been under positive selective pressure, and an antiviral activity has been described not only for human or primate SAMHD1, but also for proteins from other species, such as equine, feline, bovine, or murine SAMHD1 [42,74,80,103,119,131]. During virus–host coevolution, however, viruses have evolved various strategies to circumvent the restriction factor SAMHD1. In 2011, it became clear that lentiviruses, such as HIV-2 and several SIV strains, counteract SAMHD1 with the help of their accessory proteins Vpx and Vpr, resulting in the proteasomal degradation of SAMHD1 [54,55,56,89,96]. Another strategy to evade the dNTPase activity of SAMHD1, found in HIV-1 and other SIVs, is the use of highly efficient RTs that function at low dNTP concentrations in the presence of SAMHD1 [104,105,106]. In contrast, herpesviruses employ their conserved serine/threonine kinases to phosphorylate and inactivate SAMHD1 [73,74,75]. In addition, downregulation of SAMHD1 or its relocalization might be alternative ways to circumvent antiviral restriction [67,70,73,115,126,127,130]. The variety of the hitherto uncovered SAMHD1 countermeasures emphasizes the importance of the restriction factor SAMHD1 in antiviral immunity. Of note, some RNA viruses, such as Chickungunya or Zika virus, seem to rather benefit from SAMHD1 activity by a so far unknown mechanism, suggesting that the SAMHD1 restriction might be specifically aimed at viruses whose replication depends on dNTPs [132].

Viral antagonists are promising drug targets for the development of novel antiviral therapies. Many current antiviral therapies are based on nucleoside analogues (NA) [133]. NAs highly resemble their cellular dNTP counterparts, but lead to a premature termination of DNA synthesis, when incorporated into the viral genome as substrates for RTs and DNA polymerases. In case of HIV, the activity of SAMHD1 has been shown to increase the efficacy of nucleoside RT inhibitors (NTRI) such as zidovudine (AZT) or zalcitabine (ddC) [134,135,136]. Since NAs are not targeted by SAMHD1 or are only hydrolyzed at a significantly slower rate than cellular dNTPs, they have an increased efficacy in the presence of SAMHD1. Depletion of SAMHD1 in macrophages and CD4^+^ T cells leads to a dramatic decrease in HIV sensitivity against RT inhibitors, especially those that resemble thymidine. By degrading cellular dNTPs, SAMHD1 leads to an enhanced concentration of exogenous NAs and therefore to a more efficient viral inhibition [134,135]. Existing herpesviral NA therapies often exploit the ability of herpesviral kinases to phosphorylate their targets. Prodrugs such as ganciclovir or acyclovir, two guanosine analogues, are only activated through initial monophosphorylation by herpesviral kinases such as HCMV pUL97 or HSV TK, and are thereby highly specific to virus-infected cells [137,138,139,140]. However, since herpesviruses are known to accumulate drug-resistant mutations against nucleoside analogues, other therapeutics are needed in these cases to substitute for NA treatment. CHPK inhibitors are therefore promising candidates for alternative therapies to release the block in SAMHD1-mediated antiviral activity. The most advanced candidate, maribavir, was shown to potently reduce HCMV replication through inhibition of the viral kinase pUL97, and is currently under clinical investigation in a phase III study [141,142]. Thus, development of efficient strategies to block the various SAMHD1 evasion mechanisms such as the inhibition of viral kinases as well as SAMHD1-resistant NAs, will result in a higher efficacy and capability of antiviral therapies in the future.
